# Factors associated with early initiation of breastfeeding in central Saudi Arabia: a hospital-based survey

**DOI:** 10.1186/s13006-023-00598-6

**Published:** 2023-11-16

**Authors:** Ameinah Thamer Alrasheedi

**Affiliations:** Department of Pediatrics, Unaizah College of Medicine and Medical Sciences, Qassim University, Unaizah, Kingdom of Saudi Arabia

**Keywords:** Early initiation of breastfeeding, Al Qassim Region, BFHI, Central Saudi Arabia

## Abstract

**Background:**

Early initiation of breastfeeding is central to the success of infant feeding. The World Health Organization (WHO) therefore recommends breastfeeding within the first hour of birth and has set a target to increase the rate to 70% globally by 2030. This suggests the necessity of systematic investigations to capture trends in early initiation of breastfeeding to avail health systems of up-to-date information in the interest of appropriate policy making. Hence, this study was designed to investigate the factors associated with early initiation among mothers who delivered babies in public healthcare facilities in the Al-Qassim Region, Saudi Arabia.

**Methods:**

The research was a hospital-based, cross-sectional study which featured the recruitment of 546 mothers from March 2021 to June 2021 in five public hospitals. A questionnaire was the tool used for data collection and this was administered via face-to-face, structured interviews. Data were analyzed using binary logistic regression including unadjusted (UOR) and adjusted odds ratio (AOR) with a 95% confidence interval (CI).

**Results:**

The prevalence of early initiation of breastfeeding was 23.1% (120 of 519 respondents). Maternal and paternal socio-demographic variables, household characteristics, parity, age of the previous child, breastfeeding the previous child or otherwise and the sex of the newborn were not associated with the early initiation of breastfeeding. Mode of delivery and antenatal education about breastfeeding were significant factors. Postnatal care practices were also significant: the provision of help in positioning babies for breastfeeding (AOR 3.5; 95% CI 1.62, 7.57), 24-hour rooming-in (AOR 6.26; 95% CI 1.31, 29.8) and encouragement to practice early initiation of breastfeeding (AOR 3.05; 95% CI 1.71, 5.43) were good, better and the best factors associated with early initiation of breastfeeding respectively.

**Conclusion:**

The prevalence of early initiation of breastfeeding is poor and represents a threat to child survival in the study area. Postnatal care practices are crucial factors strongly predisposing mothers to early initiation of breastfeeding and should therefore be institutionalized in health policy frameworks to promote the same in Saudi Arabia.

## Background

Breastfeeding is the ideal and safest source of nutrition which promotes healthy growth in infants [1]. It is also considered one of the cheapest practices that foster infant survival and development [2]. Breastfeeding reduces the mortality of children under five years owing to infectious diseases and reduces maternal mortality due to breast cancer [1–3]. These and much more valuable predispositions of breastfed babies and breastfeeding mothers are further enhanced with the early initiation of breastfeeding. It is therefore logical that the World Health Organization (WHO) and United Nations Children’s Fund (UNICEF) recommend early initiation of breastfeeding within the first hour of childbirth [1]. It is the cornerstone of ideal breastfeeding which is practiced by putting newborns at the breast within the first hour of life [4].

Early onset of breastfeeding is tremendously vital to child survival. Evidence shows that the probability of death is 33% higher among newborns for whom breastfeeding initiation was delayed (i.e., when breastfeeding is attempted after one hour of a newborn’s life) when compared with those who enjoyed early initiation of breastfeeding [4, 5]. According to the WHO and UNICEF, less than half of mothers worldwide (42%) commence breastfeeding within the initial hour and subsequently practice breastfeeding exclusively [6, 7]. Therefore, the Global Breastfeeding Collective—led by the WHO and UNICEF, introduced the Global Breastfeeding Scorecard in 2017. The scorecard has seven policy action priorities, each with an indicator and a set goal to be accomplished by 2030 [7]. The aims are to encourage and document progress in breastfeeding promotion, protection, and support globally. The target is to increase the extent of early initiation of breastfeeding and exclusive breastfeeding to 70% globally.

The prevalence of early initiation of breastfeeding in the Middle East and North Africa is approximately 35% [6]. The Kingdom of Saudi Arabia (KSA) is the biggest country in the Middle East, with thirteen provinces, and is considered a high-income country. Data about breastfeeding in Saudi Arabia are inadequate and insufficient. The report of a review of seventeen cross-sectional studies focused on breastfeeding in Saudi Arabia shows that most of the studies are poorly designed [8]. Only 5 of 17 studies employed standard definitions, thereby making the comparison of findings difficult. For instance, the rate of exclusive breastfeeding could not be estimated with any degree of accuracy, with rates varying between studies from 0.8 to 43.9%. Moreover, the average duration that mothers breastfed decreased progressively with time, dropping from 13.4 months in 1987 to 8.5 months in 2010 [8]. This shows the clear need for more accurate data on breastfeeding generally and early initiation of breastfeeding in particular. Despite its importance, data about early initiation, for instance, are scarce. The few studies available describe the prevalence of early initiation of breastfeeding among different regions without considering the identification of factors associated with the same [9]. The systematic investigation of early initiation of breastfeeding and its associated factors will yield empirical data which will boost health-research-data availability in Saudi Arabia. Hence, the examination of independent factors associated with early initiation of breastfeeding is called for. This will help policymakers to optimize breastfeeding to achieve Vision 2030 of the Ministry of Health in Saudi Arabia. In this light, this study was designed to investigate the prevalence and factors associated with early initiation of breastfeeding among mothers who delivered babies in public healthcare facilities in the central region of Saudi Arabia.

## Methods

### Research design

The work was designed to be a hospital-based, non-experimental cross-sectional study. Hence, a snapshot of the prevalence and factors associated with early initiation of breastfeeding was examined among the final sample of 519 of 546 mothers who were recruited in public healthcare facilities across the central region of Saudi Arabia.

### Study area / research setting

The study area was the Al-Qassim region, located in the centre of the KSA. The region is one of the thirteen provinces of the Kingdom. The major public hospitals in the Qassim region were targeted. Five of these hospitals, located in five different cities, were selected for the study. None of these hospitals had Baby-Friendly Hospital Initiative (BFHI) certificates.

### Sample size

The Epi Info CDC calculator was used to estimate the required sample size. Using a 95% confidence level, 5% margin of error and an expected frequency of 50%, the sample size was 384. Hence, 384 was regarded as the minimum number of respondents that must be recruited for the study.

### Participant recruitment

**Fig. 1:**
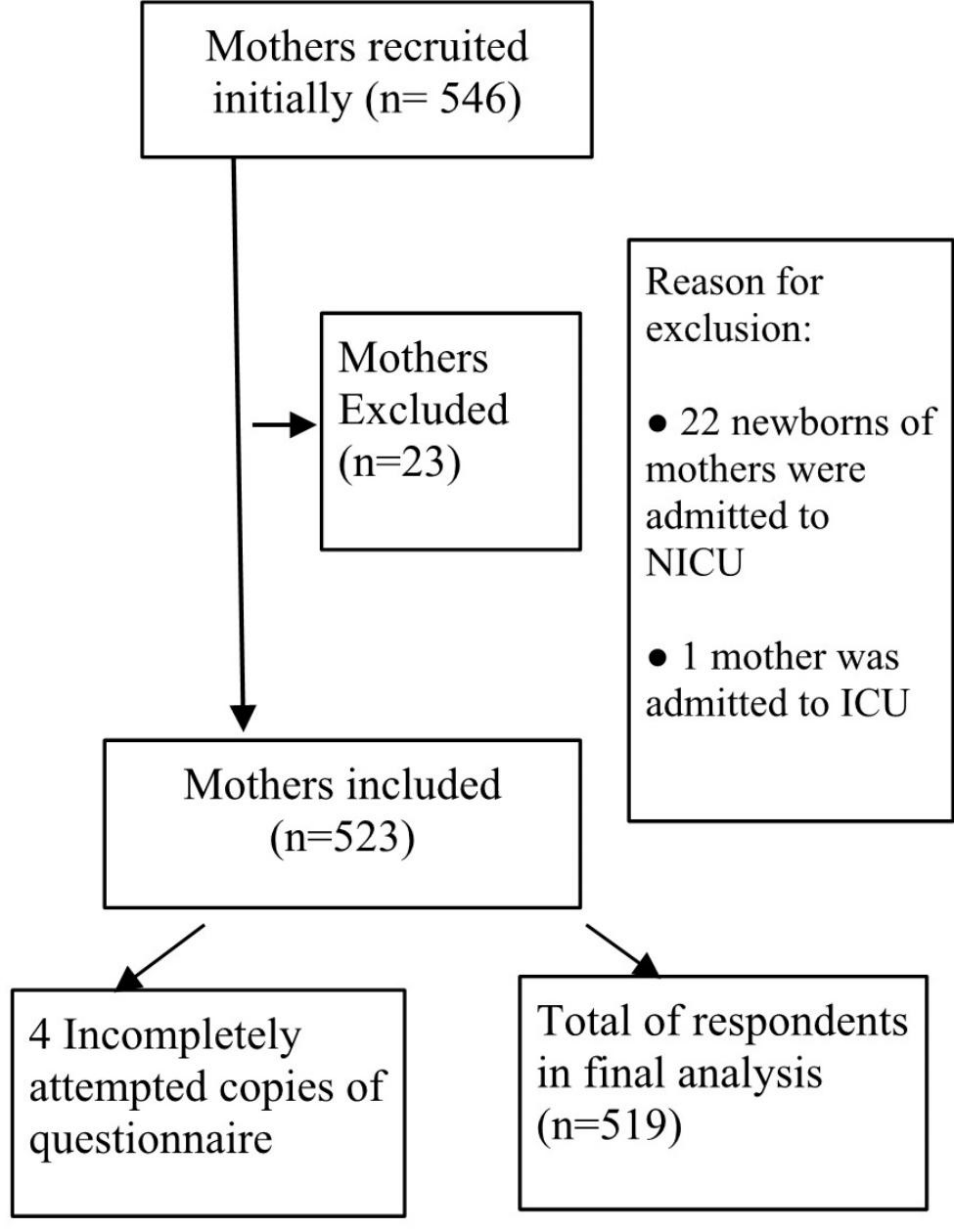
A representation of the recruitment process

Mothers who gave birth in the five selected hospitals were targeted for the study. The eligibility criteria for inclusion in the study were: age ≥ 18 years, having no history of post-postpartum complications or admission to the intensive care unit (ICU), the delivery of a singleton full-term baby (> 37 weeks), non-admission to the neonatal ICU (NICU) and newborn’s average birth weight of 2.5–4.5 kg. Saudi and non-Saudi citizens as well as primiparous and multiparous mothers were included in the study sample. A total of 546 participants were recruited, of whom 523 mothers met the inclusion criteria, but the responses of only 519 mothers were used in the final analysis. Figure 1 is a representation of the recruitment process.

### Data collection

Seven female medical interns who worked in target hospitals were trained by the principal investigator for two days about the techniques required to administer the study tool. The trainees acted as assistants in the data collection process. Mothers who gave birth in the research settings were approached after delivery and before their discharge from the hospital. The average stay of mothers who gave birth vaginally at the hospital was approximately one day, while the mothers who delivered through Cesarean section stayed for two to four days. The purpose and basic details of the research were described to them while their informed consent was sought. Once a mother agreed to participate in the study, she was asked to provide her signature on an introductory form as a way to document her informed consent. Afterwards, a research assistant administered the questionnaire via face-to-face, structured interview, under the direct supervision of the principal investigator. Data collection took four months, from March to June 2021.

### Data collection tool

A questionnaire was used for data collection. The questionnaire was structured and prepared in the English language. A pilot study was conducted among 30 new mothers in a private hospital. This was to ascertain how clear the questions were, and the length of time required to complete the questionnaire. Findings from the pilot study indicated the questions were clear because only a few questions had to be re-worded. The data obtained from the pilot study were not included in the final study results. The questionnaire was revised by three experts to ascertain face validity. The content of the questionnaire is further described under variables.

### Variables in the questionnaire

The dependent variable of the study is the early initiation of breastfeeding. This was defined as a timely (0–1 h) attempt at putting newborns to the breast after birth [1]. It was assessed by asking the participant: “How long after your child’s birth did you put him / her to the breast and he / she started breastfeeding?”.

The independent variables can be classified into six groups and were inspired by independent variables studied in relevant previous studies [10–12]. The six groups of covariates are:

1. **Maternal socio-demographic and knowledge-based characteristics**, including nationality, age, marital status, job, education, and knowledge of early initiation of breastfeeding. This knowledge was examined using a multi-choice question: “When should the breastfeeding of a new baby commence?” Responses included, “at any time during baby’s first day of life”, “within first 1 hour after delivery”, “24 hours after delivery or more”, “once baby has passed first stool”, and “I don’t know”. Respondents were finally categorized as those who know or do not know when to initiate breastfeeding based on their response.
2. **Paternal socio-demographic characteristics** including age and education.
3. **Household characteristics** including income, rural / urban residency and family support for mothers. Family support to mothers was examined with a single binary item, as follows: “Did any of your family members support or encourage you to establish breastfeeding as a preferred infant nutrition option?” Valid responses were yes or no.
4. **Antenatal experiences** including embarking on antenatal visits, and antenatal education about breastfeeding. Antenatal breastfeeding education was examined as follows: “Did you attend antenatal breastfeeding education classes that are provided by Ministry of Health (MOH) clinics at your hospital?”
5. **Maternal reproductive / breastfeeding history, mode of delivery, newborn**’**s sex**. Maternal reproductive / breastfeeding history includes parity and age of the previous child, and breastfeeding the previous child or otherwise. The mode of delivery (Cesarean section delivery versus vaginal birth) and the sex of the newborn were also independent factors in this category.
6. **Postnatal care practices**, including the encouragement to practice early initiation of breastfeeding by healthcare workers, the provision of help in positioning babies for early initiation of breastfeeding, skin-to-skin contact with babies immediately after delivery and 24-hour rooming-in.

All the independent variables were assessed nominally.

### Statistical analyses

Descriptive analyses were done using frequencies and percentages to compute the distribution of the study variables. Binary logistic regression analyses were conducted. The unadjusted odds ratio (UOR) and the 95% confidence interval (CI) were used to examine the independent factors associated with timely breastfeeding initiation. Adjusted odds ratios (AOR) with 95% CI were reported to show associated independent factors when other factors are controlled for. All statistical analyses were performed using IBM SPSS version 22.0 for Windows. Results.

### Distribution of socio-demographic characteristics and other covariates of the study

The majority of mothers (490, 94.4%) were Saudi nationals, aged from 25 to 35 years (336, 64.7%), housewives (416, 80.2%) and had University education (263, 50.7%). About two-thirds of mothers (343, 66.1%) knew about early initiation of breastfeeding while the majority of them (339, 65.3%) reported that they had family support for breastfeeding and had attended an antenatal visit (506, 97.5%). Most mothers were multiparous (398, 76.7%) while almost half of them (244, 47.0%) delivered through Cesarean section. Further details about the distributions of sociodemographic characteristics and other covariates of this study are presented in Table 1.

**Table 1:** Distribution of sociodemographic characteristics and other covariates of the study

Maternal socio-demographic characteristics and knowledge of EIBF		
Variables	Sub-groups	n (%)
Nationality	Non-Saudi	29 (5.6%)
	Saudi	490 (94.4%)
Age (years)	< 25	67 (12.9%)
	25–35	336 (64.7%)
	> 35	116 (22.4%)
Job	Housewife	416 (80.2%)
	Student	20 (3.9%)
	Paid job	83 (16.0%)
Education	Non-formal	14 (2.7%)
	Intermediate	37 (7.1%)
	Primary	38 (7.3%)
	Secondary	162 (31.2%)
	University	263 (50.7%)
	Postgraduate	5 (1.0%)
Marital Status	Married	518 (99.8%)
	Widowed	1 (0.2%)
Knowledge of early initiation of breastfeeding*	Doesn’t know when to initiate breastfeeding	168 (32.4%)
	Knows when to initiate breastfeeding	343 (66.1%)
**Paternal socio-demographic characteristics**		
**Variables**	**Sub-groups**	**n (%)**
Age (years)	20–30	77 (14.8%)
	30–40	306 (59.0%)
	40–50	108 (20.8%)
	50–60	23 (4.4%)
	> 60	5 (1.0%)
Education	Non-formal	13 (2.5%)
	Intermediate	22 (4.2%)
	Primary	18 (3.5%)
	Secondary	215 (41.4%)
	University	242 (46.6%)
	Postgraduate	9 (1.7%)
**Household characteristics**		
**Variables**	**Sub-groups**	**n (%)**
Income	< 6000 SAR [< 1600 USD]	115 (22.2%)
	6000–10,000 SAR [1600– 2666 USD]	166 (32.0%)
	10,000–15,000 SAR [2666– 4000USD]	198 (38.2%)
	15,000–20,000 SAR [4000 − 5333 USD]	37 (7.1%)
	20,000–30,000 SAR [5333– 8000 USD]	3 (0.6%)
Residency	Rural	73 (14.1%)
	Urban	446 (85.9%)
Family Support for breastfeeding**	No	32 (6.2%)
	Yes	339 (65.3%)
**Antenatal experiences**		
**Variables**	**Sub-groups**	**n (%)**
Attended antenatal visit	No	13 (2.5%)
	Yes	506 (97.5%)
Antenatal education about breastfeeding	No	413 (79.6%)
	Yes	106 (20.4%)
**Maternal reproductive / breastfeeding history, mode of delivery and newborn’s sex**		
**Variables**	**Sub-groups**	**n (%)**
Parity	Multiparous	398 (76.7%)
	Primiparous	121 (23.3%)
Age of the previous child (years)	1–2	122 (23.5%)
	3-5	181 (34.9%)
	> 5	95 (18.3%)
	No child	121 (23.3%)
Mother has previous breastfeeding experience***	No	40 (7.7%)
	No child	121 (23.3%)
	Yes	356 (68.6%)
Mode of delivery	Cesarean section	244 (47.0%)
	Vaginal	275 (53.0%)
Sex of baby	Female	244 (47.0%)
	Male	275 (53.0%)
**Postnatal care practices**		
**Variables**	**Sub-groups**	**n (%)**
Encouragement to practice EIBF	No	383 (73.8%)
	Yes	136 (26.2%)
Provision of help in positioning baby for EIBF	No	443 (85.4%)
	Yes	76 (14.6%)
Skin-to-skin contact after delivery	No	257 (49.5%)
	Yes	262 (50.5%)
24-hour rooming-in	No	43 (8.3%)
	Yes	476 (91.7%)

*Missing: 8, 1.54%; **Missing: 148, 28.52%; ***Missing: 2, 0.39%

*EIBF* early initiation of breastfeeding

### Early initiation of breastfeeding among respondents

Findings indicate that about a quarter (120, 23.1%) of mothers commenced breastfeeding within the first hour of childbirth.

### Bivariate and multivariate analyses of the factors associated with early initiation of breastfeeding

In the initial unadjusted model of the factors associated with early initiation of breastfeeding, maternal socio-demographic variables including nationality, age, job and education were not significant. Paternal socio-demographic variables including age and education were also not significant. Household characteristics including income, rural / urban residency, and family support to mothers as well as attending antenatal visits were also not significant. Maternal reproductive / breastfeeding history, including parity and age of the previous child, breastfeeding the previous child or otherwise and the sex of the newborn were also not significantly associated with early initiation of breastfeeding. The details of the results obtained from these bivariate analyses are shown in Table 2.

**Table 2:** Unadjusted models of the factors associated with early initiation of breastfeeding

Variables	Sub-groups	Early initiation birth (0-1 h)	Late initiation (> 1 h)	Unadjusted model	
		(n = 120)	(n = 399)	OR (95% CI)	*P*-value
Nationality	Non-Saudi	11 (9.2%)	18 (4.5%)	2.14 (0.98, 4.66)	0.056415
	Saudi	109 (90.8%)	381 (95.5%)	**1**	**REF**
Age (years)	25–35	80 (66.7%)	256 (64.1%)	1.26 (0.75, 2.13)	0.37878
	< 25	17 (14.2%)	50 (12.5%)	1.37 (0.67, 2.81)	0.38292
	> 35	23 (19.2%)	93 (23.3%)	**1**	**REF**
Job	Housewife	96 (80.0%)	320 (80.2%)	1.08 (0.61, 1.92)	0.78302
	Student	6 (5.0%)	14 (3.5%)	1.55 (0.52, 4.60)	0.43187
	Paid job	18 (15.0%)	65 (16.3%)	**1**	**REF**
Education	Non-formal	5 (4.2%)	9 (2.3%)	2.15 (0.69, 6.68)	0.18555
	Intermediate	11 (9.2%)	26 (6.5%)	1.64 (0.76, 3.52)	0.20691
	Postgraduate	0	5 (1.3%)	**N/A**	**N/A**
	Primary	8 (6.7%)	30 (7.5%)	1.03 (0.45, 2.38)	0.94091
	Secondary	42 (35.0%)	120 (30.1%)	1.35 (0.85, 2.15)	0.19739
	University	54 (45.0%)	209 (52.4%)	**1**	**REF**
Knowledge of early initiation of breastfeeding	Missing data	1 (0.8%)	7 (1.8%)	0.36 (0.04, 2.98)	0.34528
	Doesn’t know when to initiate breastfeeding	22 (18.3%)	146 (36.6%)	**0.38 (0.23, 0.63)**	**0.00020**
	Knows when to initiate breastfeeding	97 (80.8%)	246 (61.7%)	**1**	**REF**
Paternal age (years)	20–30	14 (11.7%)	63 (15.8%)	0.33 (0.05, 2.19)	0.25221
	30–40	69 (57.5%)	237 (59.4%)	0.44 (0.07, 2.67)	0.36943
	40–50	30 (25.0%)	78 (19.6%)	0.58 (0.09, 3.63)	0.55752
	50–60	5 (4.2%)	18 (4.5%)	0.42 (0.05, 3.22)	0.40148
	> 60	2 (1.7%)	3 (0.8%)	**1**	**REF**
Paternal education	Non-formal	4 (3.3%)	9 (2.3%)	1.35 (0.4, 4.54)	0.62907
	Intermediate	5 (4.2%)	17 (4.3%)	0.89 (0.32, 2.52)	0.82955
	Postgraduate	2 (1.7%)	7 (1.8%)	0.87 (0.18, 4.29)	0.86070
	Primary	4 (3.3%)	14 (3.5%)	0.87 (0.27, 2.73)	0.80713
	Secondary	45 (37.5%)	170 (42.6%)	0.8 (0.52, 1.25)	0.32763
	University	60 (50.0%)	182 (45.6%)	**1**	**REF**
Family income	10,000–15,000 SAR [2666-–4000 USD]	49 (40.8%)	149 (37.3%)	0.89 (0.53, 1.5)	0.66586
	15,000–20,000 SAR [4000–5333 USD]	5 (4.2%)	32 (8.0%)	0.42 (0.15, 1.18)	0.10148
	20,000–30,000 SAR [5333–8000 USD]	0	3 (0.8%)	**N/A**	**N/A**
	6000-–10,000 SAR [1600–2666 USD]	35 (29.2%)	131 (32.9%)	0.72 (0.42, 1.26)	0.25452
	< 6000 SAR [< 1600 USD]	31 (25.8%)	84 (21.1%)	**1**	**REF**
Residency	Rural	19 (15.8%)	54 (13.5%)	1.2 (0.68, 2.12)	0.52564
	Urban	101 (84.2%)	345 (86.5%)	**1**	**REF**
Family support for breastfeeding	Missing data	34 (28.3%)	114 (28.6%)	1 (0.63,1.58)	0.99310
	No	8 (6.7%)	24 (6.0%)	1.12 (0.48, 2.58)	0.79868
	Yes	78 (65.0%)	261 (65.4%)	**1**	**REF**
attended antenatal visit	No	6 (5.0%)	7 (1.8%)	2.95 (0.97, 8.94)	0.056
	Yes	114 (95.0%)	392 (98.3%)	**1**	**REF**
Antenatal education about breastfeeding	No	87 (72.5%)	326 (81.7%)	**0.59 (0.37, 0.95)**	**0.029**
	Yes	33 (27.5%)	73 (18.3%)	**1**	**REF**
Parity	Multiparous	97 (80.8%)	301 (75.4%)	1.37 (0.83, 2.28)	0.221
	Primiparous	23 (19.2%)	98 (24.6%)	**1**	**REF**
Age of the previous child (years)	1–2	36 (30.0%)	86 (21.6%)	1.39 (0.82, 2.33)	0.219
	3–5	42 (35.0%)	139 (34.8%)	**1**	**REF**
	> 5	19 (15.8%)	76 (19.1%)	0.83 (0.45, 1.52)	0.542
	No child	23 (19.2%)	98 (24.6%)	0.78 (0.44, 1.37)	0.385
Breastfeeding history (breastfeeding of the previous child)	Missing data	1 (0.8%)	1 (0.3%)	0.97 (0.1, 9.45)	0.979
	No	5 (4.2%)	35 (8.8%)	0.42 (0.16, 1.09)	0.075
	No child	23 (19.2%)	98 (24.6%)	0.7 (0.42, 1.17)	0.169
	Yes	91 (75.8%)	265 (66.4%)	**1**	**REF**
Sex of new baby	Female	62 (51.7%)	182 (45.6%)	1.27 (0.85, 1.92)	0.244
	Male	58 (48.3%)	217 (54.4%)	**1**	**REF**
Skin-to-skin contact after delivery	No	41 (34.2%)	216 (54.1%)	1	**REF**
	Yes	79 (65.8%)	183 (45.9%)	**2.27 (1.49, 3.48)**	**0.0110**

*CI* confidence interval, *EIBF* early initiation of breastfeeding, *N/A* non applicable, *OR* odds ratio

Further, findings from the initial unadjusted model show that mothers who had no knowledge of early initiation of breastfeeding (UOR 0.38; 95% CI 0.23, 0.63) and who did not receive antenatal education about breastfeeding (UOR 0.59; 95% CI 0.37,0.95), were significantly less likely to initiate breastfeeding early. In addition, mothers who reported that they had skin-to-skin contact with their babies immediately after delivery were significantly more likely to initiate breastfeeding early (UOR 2.27; 95% CI 1.49, 3.48). The details of the results obtained from these bivariate analyses are also shown in Table 2.

The initial, unadjusted analyses of data further show that mothers who delivered their babies through Cesarean section were significantly less likely to initiate breastfeeding early (UOR 0.011; 95% CI 0.003, 0.045). Those who were encouraged to practice early initiation of breastfeeding by healthcare workers (UOR 7.85; 95% CI 4.99, 12.35); who reported that 24-hour rooming-in was practiced (UOR 6.76; 95% CI 1.61, 28.35); and who acknowledged the provision of help in positioning their babies for early initiation of breastfeeding by healthcare workers (UOR 5.78; 95% CI 3.46, 9.66), were significantly more likely to initiate breastfeeding early.

In the subsequent adjusted model of the seven significant factors associated with early initiation of breastfeeding, four factors yielded a significant AOR. Hence, the initially significant odds of increased early initiation of breastfeeding on account of the mode of child delivery (AOR 0.014; 95% CI 0.003, 0.059); the practice of 24-hour rooming-in (AOR 6.26; 95% CI 1.31, 29.8); being encouraged by healthcare workers (AOR 3.05; 95% CI 1.71, 5.43); and getting help in positioning babies (AOR 3.5; 95% CI 1.62, 7.57), remained significant when other factors were controlled for. The details of results obtained from this multivariate analysis (adjusted model) are shown in Table 3.

**Table 3:** Adjusted model of the factors associated with early initiation of breastfeeding

Variables	Sub-groups	Early initiation birth (0-1 h)	Late initiation (> 1 h)	Unadjusted model		Adjusted model	
		(n = 120)	(n = 399)	OR (95% CI)	*P*-value	OR (95% CI)	*P*-value
Mode of delivery	Cesarean section	2 (1.7%)	242 (60.7%)	**0.011 (0.003, 0.045)**	**0.000**	**0.014 (0.003, 0.059)**	**0.0001**
	Vaginal	118 (98.3%)	157 (39.4%)	**1**	**REF**	**1**	**REF**
Encouragement to practice EIBF by healthcare workers	No	48 (40.0%)	335 (84.0%)	1	**REF**	1	**REF**
	Yes	72 (60.0%)	64 (16.0%)	**7.85 (4.99, 12.35)**	**< 0.0001**	**3.05 (1.71, 5.43)**	**0.000**
24-hour rooming-in	No	2 (1.7%)	41 (10.3%)	1	**REF**	**1**	**REF**
	Yes	118 (98.3%)	358 (89.7%)	**6.76 (1.61, 28.35)**	**0.0090**	**6.26 (1.31, 29.8)**	**0.021**
Provision of help in positioning baby for EIBF	No	78 (65.0%)	365 (91.5%)	1	**REF**	**1**	**REF**
	Yes	42 (35.0%)	34 (8.5%)	**5.78 (3.46, 9.66)**	**0.001**	**3.5 (1.62, 7.57)**	**0.001**

*CI* confidence interval, *EIBF* early initiation of breastfeeding, *OR* odds ratio

## Discussion

The prevalence of early initiation of breastfeeding was 23.1%. This implies that approximately one of every four new mothers initiates breastfeeding within the first hour of birth in the Qassim region of Saudi Arabia. Regional findings across Saudi Arabia indicate great variations in the prevalence of early initiation of breastfeeding. El-Gilany, Sarraf and Al-Wehady surveyed 906 mothers of newborns in AI-Hassa province, Saudi Arabia and reported that only 11.4% of study participants practiced early initiation of breastfeeding [13]. The current finding is comparable with that of Alaqeel who reported that among 140 mothers of children aged from six months to four years in Buraydah, Qassim, Saudi Arabia, only 20.3% practiced early initiation of breastfeeding [14]. Dorgham et al. conducted a survey to examine breastfeeding practices among 400 mothers whose infants were at least six months old in Taif, KSA [15]. They reported that early initiation of breastfeeding was 22% which is also similar to current findings. However, Al Juaid reported that 36% of 578 new mothers were early initiators of breastfeeding in their longitudinal study spanning six months in Western Saudi Arabia [11]. Further, Azzeh et al. reported that early initiation of breastfeeding was practiced by 38% of new mothers in the report of their study among 814 mothers in Mecca, Saudi Arabia [16]. These findings showcase the variability rather than stability in the available data regarding the early initiation of breastfeeding in the regions of Saudi Arabia.

Apart from Qassim-specific and other regional findings, national data are also comparable with current findings. In their examination of trends in nutritional practices using a nationally representative sample of 5,339 Saudi-Arabian mothers of children under the age of three years, El Mouzan et al. found that only 23.2% of new mothers practiced early initiation of breastfeeding [17]. On a contrary note, the current prevalence is lower than the prevalence reported by Ahmed and Salih [9]. Ahmed and Salih reported that early initiation of breastfeeding was 43.6% in the report of their national survey among 1700 mothers who have children less than two years old in Saudi Arabia [9]. Nevertheless, current findings pervade the notion that only about a quarter of Saudi new mothers can be regarded as early initiators of breastfeeding. Early initiation is still low and presents large room for improvement considering the 70% target of the WHO and UNICEF [7]. Presently, the prevalence of early initiation of breastfeeding contributes poorly to optimal infant nutrition and largely works against child survival in Saudi Arabia.

Current findings indicate that mothers who delivered their babies through Cesarean section are more likely to initiate breastfeeding later. This supports the notion highlighting the detrimental role of this mode of delivery in the early initiation of breastfeeding which has been reported in settings around the world [18–30] including Saudi Arabia [8, 9, 11, 13, 15, 16, 31, 32]. The notion is also evidenced by a 2017 WHO Global Survey, which found that early initiation of breastfeeding is significantly lower in women with complicated pregnancies [23]. This situation calls for greater attention to the mode of delivery. A recent study in the Al-Qassim region shows that Cesarean delivery rates were high, with 55.2% of deliveries occurring by Caesarean Sect. [33]. This suggests that the prevalent mode of child delivery is deleterious to early initiation of breastfeeding. Factors underlying the phenomenon include delayed lactation due to anesthesia, post-surgical pain, tiredness, reduced alertness, respiratory distress among babies, limited ability to suckle, physiological effects resulting in late onset of lactogenesis and poor readiness to initiation breastfeeding early [11, 15, 19, 26, 28–30, 34]. Moreover, healthcare professionals often prioritize stabilizing mothers over the initiation of breastfeeding [29, 35]. However, delayed post-Cesarean breastfeeding initiation can be prevented through the BFHI, which educates hospital medical staff to promote timely initiation of breastfeeding [26, 36, 37].

Incidentally, current findings show that postnatal care practices were essential in timely breastfeeding initiation, with mothers who were encouraged and provided help in positioning babies for early initiation of breastfeeding more likely to initiate breastfeeding early. Similarly, a contemporary study in Sudan shows the positive impact of birth attendants encouraging early initiation of breastfeeding [38]. Takahashi et al. conducted secondary data analysis using the WHO dataset of the Global Survey on Maternal and Perinatal Health [23]. Their analysis of 244,569 cases of live births shows that the lack of guidelines regarding postnatal care works against the early initiation of breastfeeding. Current findings have shown that encouraging and providing help to mothers serve as enablers that benefit the practice of early initiation of breastfeeding. Current findings show that mothers who reported the practice of 24-hour rooming-in were more likely to initiate breastfeeding early. The findings of Azzeh et al. also support the same [16]. Karim et al. similarly reported that mothers who were offered postnatal care were more predisposed to practice early initiation of breastfeeding [21]. Skin-to-skin contact between mothers and their babies was another postnatal care practice which was positively consequential for the early initiation of breastfeeding. Gayatri and Dasvarma conducted a secondary data analysis of 6616 cases using the Indonesia Demographic and Health Survey 2017 while Cozma-Petruţ et al. conducted a cross-sectional survey among 1399 Romanian mothers of children aged less than two years and found that skin-to-skin contact was a significant factor in early initiation of breastfeeding [24, 39].

All four indicators of postnatal care practices in the current study have demonstrated the significance of the motivational role of environmental conditions, which positively impact the early initiation of breastfeeding. Receiving antenatal education about breastfeeding was a significantly pertinent factor in the early initiation of breastfeeding in the current study. Similarly, Ahmed and Sahih as well as Azzeh et al. reported that having breastfeeding information and being informed about the importance of breastfeeding respectively were significantly associated with early initiation of breastfeeding [9, 16]. The effect of antenatal education on breastfeeding practice has been addressed by several Saudi-based studies which affirmed the gap between education / knowledge and the practice of breastfeeding among Saudi mothers [40–43].

The successful implementation of BFHI is evident in Gulf countries like Oman which recorded an optimal early initiation of breastfeeding rate of 71% [44], and in the UAE where the rate has risen to 59% and even up to 80% [45, 46]. Therefore, practices that are institutionalized in healthcare settings such as those recommended under the BFHI including the provision of help in positioning a baby, encouragement, the promotion of mother-child skin-to-skin contact and 24-hour rooming-in favor of early initiation of breastfeeding.

Current findings also show that family support to mothers was not a significant factor and more than a quarter of respondents (148, 28.5%) were evasive in responding to the question of whether their family supported them in breastfeeding or not. Although the majority (339, 65.3%) declared this support while a third (32, 6.2%) did not, the noticeable degree of evasiveness is probably born out of the desire to avoid “reporting” non-supporting family members. Family values are held in high regard among Saudi people and this may dampen the willingness to declare a lack of support, despite the anonymous nature of the data collection process [47]. This is worrying considering that findings have indicated family support as a factor in successful breastfeeding in similar contexts: Dashti et al. reported that the positive attitude of fathers and maternal grandmothers is a positive factor influencing the duration of breastfeeding in Kuwait [48]. In another report, Dashti et al. further reported the crucial role of fathers in initiating breastfeeding among mothers in Kuwait [49]. This highlights the relevance of looking into family support in interventions to improve early initiation of breastfeeding in Saudi Arabia.

This study has a few limitations. Firstly, causal inferences cannot be drawn from any associations found between the independent variables and early initiation of breastfeeding because a cross-sectional study design was employed. Secondly, only mothers admitted to public health facilities in the Al-Qassim region were included. Therefore, the findings are not generalizable to mothers admitted to private health facilities.

## Conclusion

The prevalence of timely initiation of breastfeeding is poor, meaning there is large room for improvement when considering the 70% target of the WHO and UNICEF. This poor prevalence is a contributor to suboptimal infant nutrition which stands against child survival in Saudi Arabia. The significant roles of knowledge of early initiation of breastfeeding, antenatal education about breastfeeding, Cesarean section rather than vaginal delivery, the provision of help in positioning babies, the encouragement to practice early initiation of breastfeeding, skin-to-skin interaction between mothers and their babies, and 24-hour rooming-in, largely point towards the relevance of the BFHI. These significant factors are elements of the BFHI the adoption and implementation of which can always be improved at the level of health care settings. The poor prevalence of early initiation of breastfeeding can be seen as a reflection of the poor state of compliance with the BFHI. It is therefore recommended that the norms of BFHI be institutionalized at hospitals.

## Data Availability

Data will be made available on reasonable request.

## Abbreviations

AOR: Adjusted odds ratio
BFHI: Baby Friendly Hospital Initiative
CI: Confidence interval
EIBF: Early initiation of breastfeeding
ICU: Intensiver care unit
KSA: Kingdom of Saudi Arabia
MOH: Ministry of Health
NICU: Neonatal intensiver care unit
UNICEF: United Nations International Childrens’ Emergency Fund
UOR: Unadjusted odds ratio
WHO: World Health Organization
